# A Rare Case of Invasive Malignant Melanoma Metastasis in the Vulvar Mucosa 11 Years After Diagnosis and Treatment

**DOI:** 10.7759/cureus.62259

**Published:** 2024-06-12

**Authors:** Bhavya Thuremella, Robyn Schultz, Sukanya Mohan, Maria Castilla

**Affiliations:** 1 General Surgery, Dr. Kiran C. Patel College of Osteopathic Medicine, NSU (Nova Southeastern University) Florida, Fort Lauderdale, USA; 2 General Surgery, Dr. Kiran C. Patel College of Osteopathic Medicine, NSU (Nova Southeastern University) Florida, Tampa, USA; 3 Osteopathic Medicine, Dr. Kiran C. Patel College of Osteopathic Medicine, NSU (Nova Southeastern University) Florida, Clearwater, USA; 4 General Surgery, HCA Florida Fawcett Hospital, Port Charlotte, USA

**Keywords:** sox 10, melan a, surgery general, gynecologic oncology surgery, mucosal melanoma metastasis, vulvar mucosal melanoma, mucosal malignant melanoma

## Abstract

Mucosal melanoma is rare and the occurrence of an invasive malignant melanoma metastasis 11 years post-initial diagnosis is equally uncommon. This is a case of a 66-year-old woman with a history of bilateral vulvar invasive melanoma, who presented with an enlarging inguinal mass with associated tenderness upon palpation. After a right inguinal excisional lymph node biopsy, the pathological findings determined the final diagnosis as metastatic melanoma. To the best of our knowledge, this is the first report of vulvar mucosal melanoma metastasis greater than 10 years after initial diagnosis and treatment in the English language. This case discusses how treatment options for metastatic mucosal melanoma pose a challenge in such cases where follow-up for medical care is lacking. It also highlights the need for further preventative techniques and research directed towards screening techniques, staging guidelines, and treatment options for mucosal melanoma.

## Introduction

Melanomas originate from melanocytes which derive from neural crest cells, located in the basal cell layer of the epithelium. In cutaneous melanomas, the primary risk factor is ultraviolet radiation exposure. Melanocytes are prone to atypical mitotic activity due to their high turnover rate and often result in the development of cancer. For cutaneous melanoma, the most prevalent demographic is Caucasian males [1]. In contrast, mucosal melanomas, which only comprise 1.3% of all melanoma cases, do not have any specific environmental risk factors known to contribute to their development [1,2]. The only risk factors that have been identified with mucosal melanomas are that they primarily affect Caucasian females in their late 60s, most commonly involving the vulvovaginal, anorectal, and head and neck regions. Some studies indicate that the occurrence of a neuroblastoma RAS viral oncogene homolog (NRAS) mutation could be associated with mucosal melanomas in the vulvovaginal, anorectal, and head and neck regions [1,3]. However, individuals with a familial history of cutaneous melanoma may have an increased risk of developing mucosal melanomas [1].

Staging and histopathological predictors are not clearly defined for mucosal melanoma, as compared to cutaneous melanoma, due to the combination of rarity and variability in presentation. For mucosal melanomas, Breslow depth, ulceration, and mitoses have not been proven to show any influence on survival [4]. Nonetheless, negative margins for surgical resections still showed significant improvement in survival rates [4].

Melanoma metastasis is a common occurrence, often manifesting shortly after the initial diagnosis. An average time of 2.2 years for melanoma metastasis [5], with mucosal melanoma patients experiencing lymph node metastasis within a shorter span of seven months [2]. However, the 10-year recurrence rate post-initial diagnosis is reported at only 2.4% [6]. The lungs are the most frequent site of distant metastasis for melanoma, while mucosal melanoma localized to areas such as the vagina commonly metastasizes to the peritoneum [5]. Melanoma, especially with mucosal involvement, is a highly lethal form of cancer, with a five-year survival rate of 25% [2].

We experienced a case with a patient who was diagnosed with vulvar melanoma, a rare type of mucosal melanoma, 11 years ago, returning with metastasis of the primary pathology. In this paper, we report the findings spanning from the initial diagnosis to today and compare them to other cases of metastatic mucosal melanoma. Verbal consent to proceed with writing this case report was obtained from the patient over the phone.

## Case presentation

**Figure 1:**
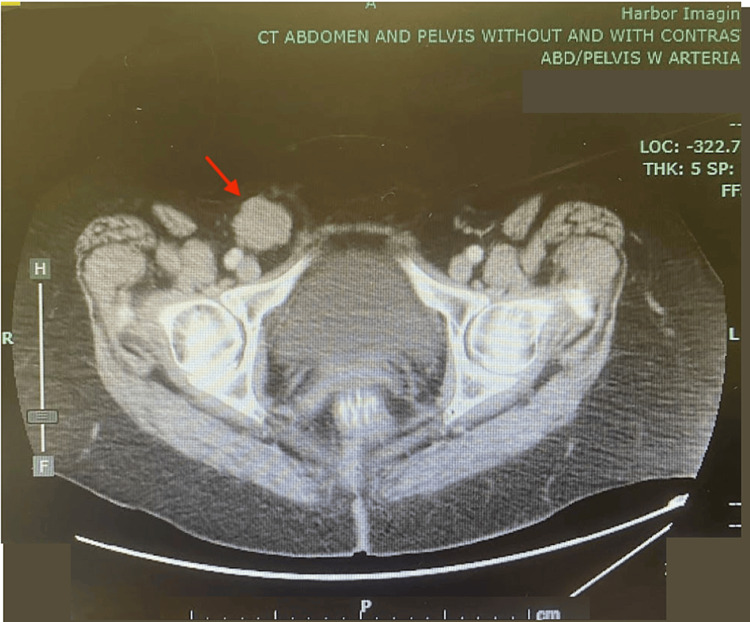
Preoperative CT of pathologic right inguinal lymph node

A 66-year-old Caucasian woman visited our surgical clinic due to a right inguinal mass that had grown in size over a span of a month. She had associated tenderness upon palpation of the area. An ultrasound of the right groin was performed prior to the visit and showed a heterogeneous mass without abnormal vascularity measuring 2.4 x 2.6 x 3.6 cm. A computed tomography of the abdomen and pelvis was performed following the ultrasound prior to the visit. The report showed a lobulated soft tissue attenuating mass in the region concerning a pathologic lymph node or neoplasm (Figure 1).

The patient had a history of vulvar-invasive melanoma, diagnosed in 2012. Bilateral vulvar biopsies were performed in July 2012. The left vulvar biopsy demonstrated tan-pigmented skin measuring 0.6 x 0.5 cm to a depth of 0.2 cm. The right vulvar lesion demonstrated irregular fragments of red-brown soft tissue measuring 1.5 x 0.8 x 0.5 cm. Vaginal reconstructive surgery was later done in February 2013. Pathology at this time showed left side vulva in-situ melanoma, left labium majus with in-situ and invasive malignant melanoma with a depth of invasion of approximately 5.9 mm, left labium minus with malignant melanoma in-situ, and right labium minus with invasive malignant melanoma with a depth of invasion of approximately 9 mm. All margins were negative for malignancy. Two right-inguinal sentinel lymph nodes, one left-inguinal sentinel lymph node, and one left-inguinal non-sentinel lymph node were identified with no malignancy. Two positron emission tomography-computed tomography (PET-CT) studies were performed over the course of a year spanning from 2012 to 2013, which showed no evidence of metastasis. The patient was followed for two years, after which she was lost to follow-up. 

**Figure 2:**
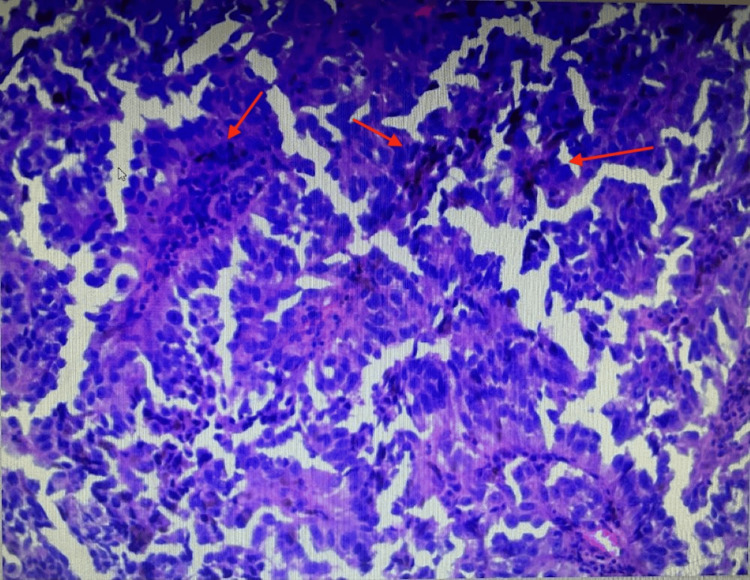
Hematoxylin and eosin staining of right inguinal lymph node biopsy with magnification 100x (positive for melanoma)

**Figure 3:**
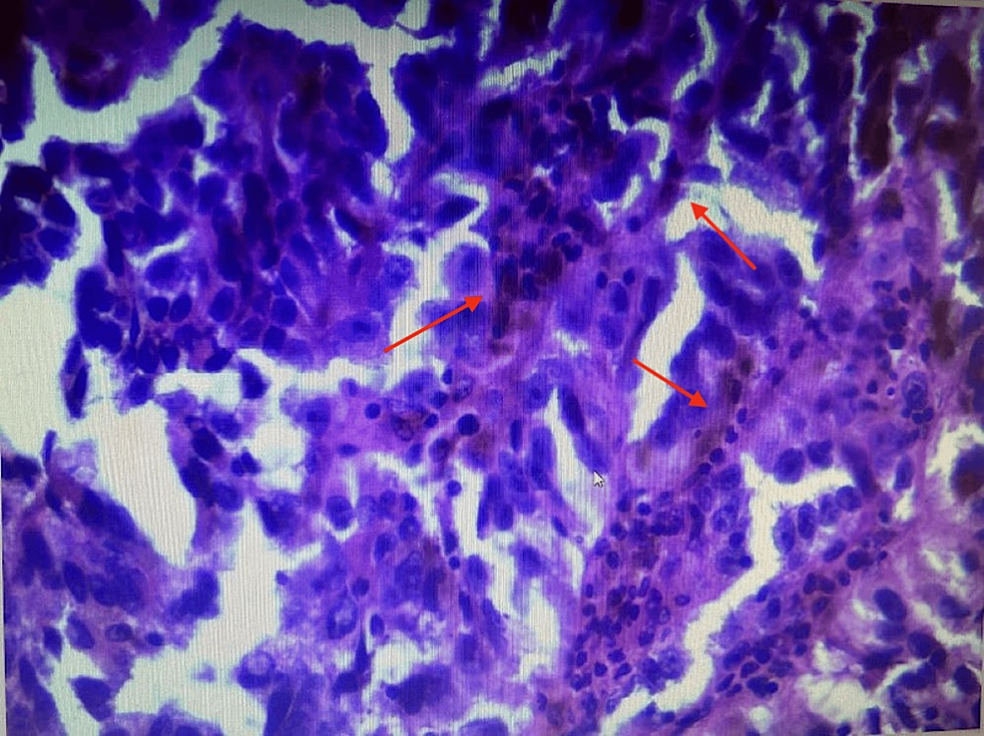
Hematoxylin and eosin staining of right inguinal lymph node biopsy with magnification 400x (positive for melanoma)

After evaluation at the clinic, the patient underwent a right inguinal excisional lymph node biopsy in October 2023. Findings included a multilobulated black subcutaneous mass. The mass was 5 x 5 x 5 cm in size. Histopathological examination of the specimen showed a spindle and epithelioid population of malignant cells with pigmentation (Figures 2-3). Immunohistochemical (IHC) staining demonstrated tumor cells positive for Melan-A (MART-1) and SRY-related HMG-box (SOX10) and negative for AE1/3 keratin, a cytokeratin. Pathological findings from the lymphatic tissue showed T cells with a decreased CD4/CD8 ratio but emphasized that this had little association with atypical cells.

Based on the pathological findings, the final diagnosis was metastatic melanoma. The patient appeared to be doing well postoperatively, with no wound site complications. She is scheduled to follow up with an oncologist to develop a treatment plan, but currently, she has no desire for additional workup postoperatively at this time.

## Discussion

Vulvar melanoma is a rare and lethal type of cancer with a typically quick progression to metastatic disease, but in this case report, we found there to be an instance of an 11-year gap in the metastatic process. No recent primary lesion was found to be contributing to this recurrence. Due to its low prevalence and minimal predisposing factors, this type of melanoma is harder to diagnose and treat. Newer screening tools such as MelaFind and SIAscope may help to determine if a biopsy is needed for patients. These devices use infrared technology and have shown improvements in specificity, sensitivity, and accuracy for biopsies [7]. MelaFind was proven to increase biopsy accuracy from 64% to 86% [7]. In our case, there was a large gap in the follow-up time from the initial diagnosis to the current presentation, which made it difficult to determine if any of these newer screening techniques could have been implemented and successful. An ideal follow-up timeline for this patient would have been every six to 12 months for five years, and then yearly exams [8].

An optimal diagnosis of melanoma occurs by performing an excisional biopsy. Methods such as the Breslow scale level help to determine the thickness and its association with the level of mitotic activity, aiding in the staging of cutaneous melanoma [9]. There is a proportional positive correlation between the Breslow level and the mitotic activity; however, Breslow depth has not been shown to influence survival for mucosal melanoma [4,9]. Another method used for staging vulvar mucosal melanoma specifically is called Chung’s classification. It has five levels and detects tumor invasion from the epithelium to expansion through the subcutaneous fat layer [10]. These staging methods help determine a proper treatment regimen for the patient.

Mutagenic markers can play important roles when diagnosing melanomas but also pose significant challenges for mucosal melanoma due to significant variability in presentation between affected individuals. Mucosal melanoma histopathological findings are most commonly positive for S-100 and vimentin, occasionally positive for human melanoma black-45 (HMB-45) and Melan-A, and usually negative for cytokeratin and epithelial membrane antigen [4]. While Melan-A is more sensitive than HMB-45, both exhibit high specificity for melanoma [7]. A retrospective analysis of primary melanomas of the oral cavity indicated that 100% of participants were positive for S-100 and HMB-45 markers, while only 57.1% tested positive for Melan-A [11]. Mucosal melanoma also frequently exhibits mutations in genes such as v-kit Hardy-Zuckerman 4 feline sarcoma viral oncogene homolog (KIT), neurofibromatosis (NF), and spliceosome factor 3b (SF3B1), with KIT specifically associated with vulvar melanoma [1]. Another mutagenic marker strongly correlated with a metastatic diagnosis is the SOX10 gene. This nuclear transcription factor demonstrates high sensitivity and specificity, particularly in cases of metastasis to lymph nodes, commonly observed in mucosal melanoma [1,12].

Earlier it was mentioned that mucosal melanoma has many mutagenic genes in comparison to cutaneous melanoma. This makes it challenging to use treatment options, such as immunotherapy, that have more success with cutaneous melanoma. However, there have been a couple of successful immunotherapy options for individuals with vulvar melanoma, including tyrosine kinase inhibitors for those with c-Kit mutations [13]. Cytotoxic T-lymphocyte-associated protein 4 (CTLA-4) and programmed cell death protein 1 (PD-1) checkpoint inhibitors have also had positive outcomes for those with metastatic vulvar melanoma [13]. Results have shown that using a combination of PD-1 and CTLA-4 inhibitors has a greater response of 37.1% versus 23.3% in PD-1 inhibitor therapy alone in patients with mucosal melanoma [14]. Other possible treatment options include undergoing chemotherapy and/or surgical resection of the region. Chemotherapy has not been proven a significant increase in longevity [1]. Surgical resection of individuals with metastatic mucosal melanoma has only been shown to increase survival rates by 30% [6].

## Conclusions

To the best of our knowledge, this is the first report of vulvar mucosal melanoma metastasis >10 years after initial diagnosis and treatment in the English language. We report a case of a 66-year-old female who presented with a right inguinal mass 11 years after the initial diagnosis. The right inguinal excisional lymph node biopsy demonstrated a 5 x 5 x 5 cm multilobulated black subcutaneous mass with histopathological findings of a spindle and epithelioid population of malignant cells with pigmentation.

In this case, we may have been able to detect the progression of the patient's melanoma better if she had followed up sooner after her vaginal reconstructive surgery in 2013. Due to this limitation, it is difficult to estimate how long it had been since the recurrence and what her prognosis might have been. This is an important case as it challenges the normal timeframe for metastasis to occur after the initial diagnosis. It further highlights the vital need for more research directed towards screening techniques and treatment options for mucosal melanoma.
